# Association between interpersonal trust and suicidal ideation in older adults: a cross-sectional analysis of 7070 subjects in Shandong, China

**DOI:** 10.1186/s12888-019-2186-4

**Published:** 2019-07-03

**Authors:** Zihang Yu, Lingzhong Xu, Long Sun, Jiao Zhang, Wenzhe Qin, Jiajia Li, Gan Ding, Qian Wang, Jing Zhu, Su Xie

**Affiliations:** 1 School of Public Health, Shandong University, Jinan, 250012 China; 2 NHC, Key Laboratory of Health Economics and Policy Research (Shandong University), Jinan, 250012 China; 3 Shandong University Center for Health Economics Experiment and Public Policy Research, Jinan, 250012 China

**Keywords:** Suicidal ideation, Interpersonal trust, Psychological distress, Older adults

## Abstract

**Backgrounds:**

Suicidal ideation is an important public health issue due to devastating mortality. In the meantime, interpersonal trust was found to be negatively associated with mental disorder and physical health. Although there is increasing evidence that interpersonal trust is a significant predictor of suicidal ideation, evidence of this association is still lacking in the developing world. The aim of this study was to test the association between interpersonal trust and suicidal ideation among older adults in China.

**Methods:**

Using a multi-stage stratified sampling strategy, 7070 older adults aged 60 and above from Shandong Province, China were recruited in this study. Socio-demographic characteristics, health status, family relationship, psychological distress, interpersonal trust and suicidal ideation in the last 12 months were obtained through face to face interviews. The association between interpersonal trust and suicidal ideation was assessed using multiple logistic regression models adjusting for socio-demographic characteristics, health status, family relationship and psychological distress.

**Results:**

7.1% of participants reported suicidal ideation in the past 12 months, which was related to gender, resident area, marital status, educational level, self-rated economic, chronic disease, self-rated health status and family relationship within 1 month, psychological distress and interpersonal trust. After adjusting for sociodemographic factors, health status, family relationship and psychological distress, interpersonal mistrust was associated with two times odds of suicidal ideation when compared to interpersonal trust

**Conclusions:**

The interpersonal trust was associated with suicidal ideation among elderly in Shandong, China. Intervention approaches regarding inducing and promoting interpersonal trust should be developed to prevent suicide.

## Background

In previous research, the factors associated with suicidal ideation can be divided into three groups: socio-demographic characteristics, psychological factors and social environmental factors [1, 2]. A large portion of literature had demonstrated the significance of a wide variety of socio-demographic and psychological causes of suicidal ideation [3, 4], while the social (interpersonal) related factors of suicidal ideation were often neglected, consequently, were not received enough attention.

Interpersonal trust, the basis of almost entire major theories of interpersonal relationships [5], was one of the most important ingredients among social environmental factors. As Giddens said, trust is faith in the reliability of other people in a specified set of events or outcomes [6]. A growing body of evidence in developed countries suggests that interpersonal trust is associated with mental disorder and physical health. For example, in the USA, a prospective study found that perceptions of higher levels of interpersonal trust are associated with lower odds of developing mental disorder [7]. Pollack found the relationship between mistrust and poorer self-rated health, depression and functional limitations [8]. In Australian, Phongsavan reported that after controlling socio-demographic characteristics and health conditions, those who have higher levels of trust were inversely associated with psychological distress [9]. In Canada, the three studies undertaken by Rotenberg indicated that loneliness scores on the revised UCLA loneliness scale were negatively correlated with interpersonal trust [10]. In developing countries, there is much less evidence indicating that interpersonal trust is linked to the health condition. In the China context, Winnie Yip’s work suggested that trust exhibited strong and consistent associations with health and well-being in the rural Chinese population [11]. Older adults who mistrust people were more likely to suffer from poor physical and mental health at the same time. These negative influences may, in turn, increase the probability that one would consider suicide. In addition, one ecological investigation conducted in 11 European countries found that social trust was inversely associated national suicide rate after controlling for other sociodemographic and psychological variables [12]. During the economic crisis, experts found the presence of a low interpersonal trust was the significant predictor of suicidal ideation and suicide attempt [13]. Previous studies on the association between suicidal ideation and trust mainly focused on teenagers, while the correlation may be stronger among seniors [14, 15]. Compared with the younger population, the elderly had a greater likelihood of cognitive impairment and disadvantaged physical condition, which would lead to more need for emotional support and material assistance that generally acquired from trusted relationships, especially when negative life events occur [16, 17]. Consequently, lacking trust would preclude them from seeking help and social support in time. There are few studies showed that trust is linked to suicidal ideation in developed economies. In Japan, Masayuki found individual-level mistrust was associated with suicidal ideation after adjusting for social support and psychological distress among the elderly living in the rural area [18]. The same result is also found that social trust accumulated reduce the probability that one will consider suicide among male living in urban areas and subjects with a mean age of 54.5 [19, 20]. In South Korea, by using a nationally representative longitudinal cohort database, Kim showed that the low interpersonal trust group has a greater likelihood of suicidal ideation [21]. Because research on interpersonal trust and suicidal ideation are still in an early stage, it is necessary to record the possible relationships.

Suicide is a major public health problem worldwide and resulted in 842,000 deaths in 2013 [22]. In China, suicide accounted for 3.6% of general population deaths and ranked as the fifth most important cause of death [23]. The suicide rate was 9.8 per 100 thousand people in the late 2010s in China, demonstrating a significant decline trend compared with 23 per 100 thousand people in the late 1990s [23, 24]. However, the decline has not been found among older adults, on the contrary, the rate in elderly was strikingly increased and ranged from 44.3 to 200.0 per 100 thousand which is four to five times higher than average population [25]. Population aging is rapidly developing in the world, especially in China. It is estimated that the percentage of people in China aged 60 years and above will increase from 12.4% (168 million) in 2010 to 28% (402 million) in 2040 [26]. Given the alarming population aging development speed, suicide among seniors is becoming one of the most noteworthy issues. Suicidal ideation as the intermediate condition between a sound physical condition and death by suicide is an initial and inevitable stage in the process of suicide [27]. The available evidence showed that the most effective measure to prevent suicide was reducing the likelihood of suicidal ideation [2]. Therefore, even if individuals do not conduct the suicidal act, the occurrence of suicidal ideation is worth priority to study.

The current study designed to test the association between interpersonal trust and elderly suicidal ideation in China. We hypothesized that interpersonal trust would be inversely and dependently associated with suicidal ideation even after controlling for other defined risk factors.

## Methods

### Study design and data collection

Our data were collected from the Survey of the Shandong Elderly Family Health Service. The detailed sampling and quality assurance measures have been described in a previously published paper [28]. We chose Weihai, Weifang, and Heze as the investigation sites, because these three cities not only respectively represent the high, media, low level of gross domestic product (GDP) per capita (2016) in Shandong Province, but also are separately located in the eastern, central and western regions. We used a multi-stage, stratified sampling method to select a representative sample of people aged 60 years or above in the general population. In total, 7088 older adults were recruited into the study and 18 were excluded for incomplete data. In total, 7070 individuals were included in the final sample. After verbal informed consent was obtained from interviewees, all participants were interviewed individually using a structured questionnaire.

The survey group consists of 1 professor, 2 lecturers, 2 doctoral students and 28 master students from Shandong University School of Public Health. Most students major in social medicine and health service management, while others major in epidemiology, health toxicology, occupational and environmental health. To ensure the quality of the survey, first, some investigators were organized to conduct a preliminary survey in a village in caishi town of Jinan after the first draft of the questionnaire. We revised and perfected the questionnaire according the problems in the investigation process. Before the formal survey, all investigators got a survey manual and received professional face-to-face interview training to better understand the background, purpose, methods, contents and other aspects. The possible problems in the investigation were envisaged and related solutions were proposed. Then, during the investigation, the investigators were divided into six groups. After random sampling, both centralized and household survey way was adopted. Emphasis is placed on the objectivity of the investigators without inductive inquiry and subjective conjecture, and the elderly with poor understanding ability would be properly explained by interviewers. If the two sides cannot understand each other’s meaning due to dialect problems, the local staff would translate for them. Last, completed questionnaires were carefully checked by investigators, group leader and quality supervisors at the end of each day to fill and correct the missing or contradictory items. Group meetings were held as necessary to discuss the problems encountered in the investigation and propose solutions.

## Measures

### Suicidal ideation

The dependent variable of this study was suicidal ideation in the past year before the survey. We evaluated suicidal ideation through the yes/no response to the question, “have you ever seriously thought about committing suicide” if the participant answered yes, we then ask, “have you seriously thought about committing suicide at some point in the past 12 months?” We grouped suicidal ideation as “yes” (coded = 1) when subjects answer “yes” about the second question. Likewise, we categorized suicidal ideation as “no” (coded = 0) when the response was “no”. U. S National Comorbidity Survey and National Comorbidity Survey Replication have also used this question to estimate suicidal ideation [29].

### Interpersonal trust

In this paper, interpersonal trust was examined through one question, “Do you think that the people around you are trustworthy?”, respondents could answer “Yes” (coded as trust), “Depends” (coded as median trust), “No” (coded as mistrust).

### Socio-demographic factors

Age was categorized into 60–64 years old, 65–69 years old, 70–74 years old, 75–79 years old and 80 years old and above. To date, findings related to age have been mixed. We wonder which age group the elderly has a greater likelihood of suicidal ideation. Therefore, it is necessary to indicate the suicidal ideation of the elderly in different age group. Location of residence was categorized into rural, town and urban. Living arrangement and chronic diseases were binary classifications. Based on the distribution of participants, we categorized marital status into two groups: single and couple [30]. We collapsed the unmarried, divorced, and widowed to single. Education was categorized into no schooling, primary school, junior school and above. The respondents were asked to rate their economic status with four options: wealthy and not worried about livelihood, not wealthy but not worried about livelihood, not wealthy and worried about livelihood, poor and worried about livelihood. Self-rated health status was assessed by asking the participants rate their health condition with a single question: “would you say that your health has on the whole been excellent, good, fair, poor, or very poor in a month?” This five-point scale was categorized into good, fair and poor health. We ask participants “how you feel about the relationship with your family in a month?” and the answer was categorized into good, fair and poor.

### Psychological distress

Kessler Psychological Distress Scale 10 (K10) was used to assess psychological distress [31]. The reliability and validity of the scale has been proved for Chinese populations [32]. There has a validation study showed that the K10 seems to be composed of depression factor and anxiety factor and has a reasonable psychometric property [33]. This instrument has ten items and each of them having a five-point Likert response ranging from none of the time to all of the time. The total scores range from 10 to 50, with higher scores manifest higher psychological distress. The K10 total score has been showed to be a clinically useful indicator of the existence of suicidal ideation [34]. The α reliability coefficient was 0.92 in the current study.

### Statistical analysis

First, we presented descriptive statistics on the demographic characteristics and prevalence. The results were exhibited as number and percentage for categorical variables, for continuous variables as means and standard deviations (SDs). Second, the Chi-square test was performed to compare the prevalence of suicidal ideation across different groups of the respondents and the continuous variable was compared by Student’s t-test. Last, multiple logistic regression analyses were used to examine the relationship between interpersonal trust and the likelihood of suicidal ideation in the past 12 months. In model 1, we adjusted for sociodemographic characteristics, health status and family relationship to estimate the effect of interpersonal mistrust on suicidal ideation risk. In model 2, we added psychological distress to our model in order to examine how much of the variance in this association was explained by mental factor. Non-significant variables from the univariate analyses were excluded. Sample weight was included in the models. We used SPSS 22.0 to perform all statistical analyses. Associations were determined by odds ratios (ORs) and 95% confidence intervals (CIs).

## Results

### Socio-demographic characteristics of participants

Distribution of the study participants and prevalence of suicidal ideation are presented in Table 1. The study included 7070 elderly people with a mean age of 69.81 years (SD = 6.45), of which 40.3% are men and 59.7% are women. In addition, the major elderly had the following characteristics: living in rural (70.6%), not living alone (85.5%), couple (81.3%), being a primary educational level (41.4%), reporting not worried about livelihood even not wealthy (69.6%), having at least one chronic disease (68.9%), reporting good or above health (65.3%) and having a good relationship with family (94.7%) in 1 month. 87% of participants reported that they had the complete trust of those around them, 11.3% reported media trust and only 1.7% reported mistrust. The mean score of Kessler 10 was 15.34 (SD = 6.68). Of all respondents, 499 (7.1%) experienced suicidal ideation in the past 12 months. Most social demographic variables showed differences except living arrangement. There was a significant difference in suicidal ideation regarding to gender (*p* < 0.001), residence (*p* < 0.001), marital status (*p* < 0.001), educational level (*p* < 0.001), reported economic level (*p* < 0.001), chronic diseases (*p* < 0.001), self-rated health (p < 0.001) and the relationship with family (*p* < 0.001) in 1 month. Compared with the reference group, individuals who are female, aged above 75, rural resident, single, illiterate, poor and worried about livelihood, poor self-reported health and family relationship in 1 month were associated with a greater risk of suicidal ideation. The rate of people who have suicidal ideation in the past 12 months was significantly lower in participants with complete trust (6.1%) than those mistrust (23.5%) (*p* < 0.001). Regarding psychological distress, those with suicidal ideation scored significantly higher on the K10 than the counterpart (t = − 25.254, *p* < 0.001).

**Table 1:** Prevalence of suicidal ideation in the past year by participant characteristics among elderly in Shandong, China (*n* = 7070)

		Suicidal Ideation in The Past Year			
	N (%)	No (%)	Yes (%)	χ^2^/t	*p* value
Total	7070	6571 (92.9)	499 (7.1)		
Gender				30.025	0.000
Male	2846 (40.3)	2703 (95.0)	143 (5.0)		
Female	4224 (59.7)	3868 (91.6)	356 (8.4)		
Age				9.274	0.055
60–64	1577 (22.3)	1448 (91.8)	129 (8.2)		
65–69	2129 (30.1)	1970 (92.5)	159 (7.5)		
70–74	1780 (25.2)	1657 (93.1)	123 (6.9)		
75–79	975 (13.8)	923 (94.7)	52 (5.3)		
≧80	609 (8.6)	573 (94.1)	36 (5.9)		
Residence				28.204	0.000
Rural	4990 (70.6)	4586 (91.9)	404 (8.1)		
Town	524 (7.4)	503 (96)	21 (4)		
Urban	156 (22)	1482 (95.2)	74 (4.8)		
Living alone				0.116	0.733
Yes	1026 (14.5)	951 (92.7)	75 (7.3)		
No	6044 (85.5)	5620 (93)	424 (7)		
Marital status				17.342	0.000
Single ^a^	1331 (18.8)	1202 (90.3)	129 (9.7)		
Couple	5739 (81.2)	5369 (93.6)	370 (6.4)		
Educational level				48.624	0.000
No schooling	2270 (32.1)	2045 (90.1)	225 (9.9)		
Primary school	2924 (41.4)	2734 (93.5)	190 (6.5)		
Junior school and above	1876 (26.5)	1792 (95.5)	84 (4.5)		
Self-rated economic status				303.471	0.000
Wealthy and not worried about livelihood	1618 (22.9)	1557 (96.2)	61 (3.8)		
Not wealthy but not worried about livelihood	4920 (69.6)	4510 (93.7)	310 (6.3)		
Not wealthy and worried about livelihood	478 (6.8)	374 (78.2)	104 (21.8)		
Poor and worried about livelihood	54 (0.8)	30 (55.6)	24 (44.4)		
Chronic disease				49.681	0.000
No	2200 (31.1)	2115 (96.1)	85 (3.9)		
Yes	4870 (68.9)	4456 (91.5)	414 (8.5)		
Self-rated health status in one month				350.75	0.000
Good	4615 (65.3)	4443 (96.3)	172 (3.7)		
Fair	1657 (23.4)	1503 (90.7)	154 (9.3)		
Poor	798 (11.3)	625 (78.3)	173 (21.7)		
Family relationship in one month				241.192	0.000
Good	6694 (94.7)	6286 (93.9)	408 (6.1)		
Fair	310 (4.4)	250 (80.6)	60 (19.4)		
Poor	66 (0.9)	35 (53)	31 (47)		
Interpersonal trust				84.618	0.000
Trust	6154 (87)	5777 (93.9)	377 (6.1)		
Median	797 (11.3)	703 (88.2)	94 (11.8)		
Mistrust	119 (1.7)	91 (76.5)	28 (23.5)		
Psychological distress (M ± SD)	15.34 ± 6.68	14.62 ± 5.90	24.78 ± 8.84	25.254	0.000

Note: ^a^ Others included those who are unmarried (0.9%), widowed (17.6%), or divorced (0.2%)

### Association between interpersonal trust and suicidal ideation in the past 12 months

Table 2 shows the results of multiple logistic analysis in two models. In model 1, interpersonal mistrust was associated with a statistically significant increase in likelihood of suicide ideation (β = 1.20; OR = 3.33; 95%CI 2.00–5.54). The results in model 2 indicated that controlling for psychological distress slightly attenuated the positive association between interpersonal mistrust and suicide ideation (β = 0.72; OR = 2.06; 95%CI 1.5–3.70) but it still remained statistically significant. As for other characteristics, running all significant covariates simultaneously within multivariate models reveal that the independent influence factors for suicidal ideation were gender, education, self-rated economic and health status and relationship with family.

**Table 2:** Associations between interpersonal trust and suicidal ideation in the past year among elderly in Shandong, China

Variables	Model 1			Model 2		
	β	OR	95% CI	β	OR	95% CI
Gender						
Male ^Ref^		1.00			1.00	
Female	0.55	1.73***	1.38–2.17	0.33	1.39**	1.09–1.77
Residence						
Rural ^Ref^		1.00			1.00	
Town	−0.65	0.52**	0.33–0.84	−0.49	0.61	0.37–1.00
Urban	−0.20	0.82	0.61–1.11	−0.08	0.92	0.67–1.27
Marital status						
Single ^a; Ref^		1.00			1.00	
Couple	− 0.23	0.80	0.64–1.00	−0.16	0.85	0.67–1.09
Educational level						
No schooling ^Ref^		1.00			1.00	
Primary school	−0.15	0.86	0.68–1.08	− 0.32	0.73**	0.57–0.92
Junior school and above	−0.30	0.74	0.55–1.01	−0.57	0.57***	0.41–0.79
Self-rated economic status						
Wealthy and not worried about livelihood ^Ref^		1.00			1.00	
Not wealthy but not worried about livelihood	0.19	1.21	0.90–1.64	−0.06	0.94	0.69–1.30
Not wealthy and worried about livelihood	1.20	3.31***	2.28–4.82	0.69	2.00**	1.34–3.00
Poor and worried about livelihood	1.76	5.82***	2.98–11.40	1.16	3.18**	1.51–6.69
Chronic disease						
No ^Ref^		1.00			1.00	
Yes	−0.37	0.69**	0.53–0.89	−0.23	0.80	0.61–1.04
Self-rated health status in one month						
Good ^Ref^		1.00			1.00	
Fair	0.63	1.87***	1.47–2.37	0.27	1.32*	1.02–1.69
Poor	1.44	4.23***	3.29–5.48	0.45	1.57**	1.18–2.10
Family relationship in one month						
Good ^Ref^		1.00			1.00	
Fair	0.71	2.04***	1.46–2.85	0.16	1.18	0.82–1.70
Poor	1.69	5.41***	3.08–9.48	0.86	2.36**	1.26–4.41
Interpersonal trust						
Trust ^Ref^		1.00			1.00	
Median	0.46	1.59***	1.22–2.07	0.11	1.12	0.84–1.50
Mistrust	1.22	3.38***	2.04–5.62	0.73	2.08*	1.56–3.73
Psychological distress (M ± SD)				0.13	1.14***	1.13–1.16

^a^ Others included those who are unmarried (0.9%), widowed (17.6%), or divorced (0.2%); ^Ref^ denotes reference category; * *p* ≤ 0.05, **p ≤ 0.01 and ***p ≤ 0.001

## Discussion

In the current study, we investigated the association between interpersonal trust and suicidal ideation in a large population-based sample of older Chinese. We found strong evidence of an increased risk of suicidal ideation among seniors reporting interpersonal mistrust. We also observed the prevalence of elderly suicidal ideation was 7.1%. In addition, suicidal ideation was also found significantly correlated with gender, education, economic status, physical health, and family relationship. Some associations were attenuated after controlling for psychological distress, emphasizing the importance of mental health.

Our study examined the relationship between interpersonal trust and suicidal ideation both with and without psychological distress included in the model. After controlled other variables, such as gender, age, residence, marital status, education, self-rated economic and health condition, chronic diseases and relationship with family, both models exhibited similar result that the odds of suicidal ideation increased when the elderly report they mistrust people around. This finding here emphasis the intervention designed specifically to induce and promote trust may be effective in reducing suicidal ideation. As the autonomous organization in China, community residents’ committees and village committee should provide more activities according to the needs of the elderly, so that older adults can establish their trust and emotional foundation at the same time as they participate. At the same time, there is evidence that informal social networks like having high neighborhoods attachment usually establish higher interpersonal trust than engaging formal civic participation [35]. The main purpose in including the psychological distress in Model 2 is to see how the OR value would change when compared to Model 1. The OR value of interpersonal mistrust was remarkably reduced from 3.33 in Model 1 to 2.06 in Model 2. Previous research showed that people who report low level of interpersonal trust had higher odds ratio of psychological distress and people with psychological distress were more likely to have suicidal ideation [36]. Therefore, adjusting psychological distress rendered the influence of interpersonal trust less prominent.

The prevalence of suicidal ideation in the past year among the older people in current study was 7.1%, which was similar to the 7.3% found in general population of United States [37]. However, due to geography and socioeconomic background in various studies, the incidence rates are also varied in a large range. Several studies have documented the considerable gap in the incidence of suicidal ideation in China. For example, a study conducted in Hunan, located in south central area in China, reported that 14.5% of elderly (60+) lived in rural communities was experienced suicidal ideation in the last 12 months and the figure down to 14.1% after standardization [2]. Another study conducted in Shanghai and Beijing reported the prevalence estimate was 3.1% [38]. The difference with our research might due to geography since the study sample in the research carried out in Hunan and metropolitan cities were respectively strictly limited to the rural residents and urban residents, while our sample included both rural and urban populations in Shandong for the better representation. Numerous studies have shown that the suicide rate in the rural population is higher than those in urban population in China [23, 39]. The figure in previous studies was 2.23% in Japan for aged 65 and above and 3.3% in America with data collected from 2001 to 2003 National Comorbidity Survey Republication [29, 40]. Both are lower than the percentage in our study. One explanation might be that due to China’s booming economy and socioeconomic changes in recent decades, the older people have to bare unfamiliar challenges and encounter various problems which make them vulnerable to negative emotions like anxiety and depression and led to the desire to escape through suicide.

Being female, low educational level, and self-rated poor health condition was found to be positively significantly correlated with senior’s suicidal ideation in the current study. These influences have been repeatedly emphasized in previous studies as being important elderly risk indicators [41–44]. Besides, relationship with family also plays a role in elderly suicidal ideation especially for Chinese family. Bad family relationship was positively correlated with the occurrence of suicidal ideation. The result is consistent with those conducted among Asian Americans [45]. These results highlight the importance of establishing harmonious family especially among young-old elderly for suicide prevention.

In our study, we also found that poverty and worried about livelihood was the strongest significant predictor of suicidal ideation. The result is supported by a study collected data from the National Health and Nutrition Examination Survey which demonstrated that income to poverty ratio was one of the most important correlates for suicidal ideation [46]. The majority of seniors with self-rated poor financial state especially who unable to guarantee the basic life have to undergo much more problem such as physical illness, mental disorder and lack of social support which would further produce wish to commit suicide. Although mood disorder such as anxiety and particularly depression has been repeatedly proved to be the key risk factor in high-income countries, an impulse-control disorder caused by economic distress and discordant family relations is the strongest predictor in low-and-middle income countries particularly China [44]. China has been proved to be one of the countries which have the low prevalence of mental illness among suicide victims [47]. This may be the reason why psychological distress measured by K10 happened not to be the strongest predictor in this study. Therefore, suicide intervention for older people should pay more attention to economic status and not be restricted to psychological interventions.

### Strengths and limitations

This study had some limitations. First, although the items are commonly used by existing studies [21], one item measure of suicidal ideation and interpersonal trust is less reliable and accuracy than more detailed measurement, which cannot exclude the possibility of measurement error. Suicidal ideation could be biased reporting by having different interpretations of what “serious” means. Interpersonal trust may be under-reporting biased in consideration of interpersonal harmony is the core value of Chinese culture. Another limitation of the measure of interpersonal trust is that the elderly were not asked to consider different groups, like family, neighbors, friends. However, we believe that as long as there is a trusted person, whether this person is relatives or neighbors, the elderly could obtain benefits of trust which include reducing the possibility of depression and loneliness and improving health. Second, it is possible that suicidal ideation shapes report of interpersonal trust. Seniors with suicidal ideation may be preoccupied with negative thinking and may misinterpret others as untrustworthy. The cross-sectional survey design does not permit the determination of the cause-effect relationship of suicidal ideation. Third, subjective measures instead of objective information might be subject to seniors’ recall bias. Finally, there may be other confounding factors than those available for consideration in this study. Despite these limitations, by using data from a large size of the sample, our study was given a high degree of statistical power. As far as we know, this is the first study to investigate the association between suicidal ideation and interpersonal trust among Chinese elderly. This study extends the literature about the role interpersonal trust plays in suicidal ideation and provides additional targets for interventions to prevent suicide in older adults. Future research about interpersonal trust can focus on its clinical application in suicide risk assessment.

## Conclusions

Our findings revealed that interpersonal mistrust is a possible risk factor for suicidal ideation. Besides, gender, education, self-rated economic status, self-rated health status and relationship with family in 1 month were significantly associated with suicidal ideation in the past 1 year. The results of this study suggested that multi-faceted interventions for seniors including activities which can establish trust would be conductive to reduce the risk of suicidal ideation.

## Data Availability

The datasets supporting the conclusions of this article are available from the corresponding author on reasonable request.

## Abbreviations

CI: confidence interval
K10: Kessler Psychological Distress Scale 10
OR: odds ratio
SD: standard deviation
